# Loss of Adenosine Deaminase Acting on RNA 1 Induces Panoptosis and Immune Response in Ulcerative Colitis Gut Mucosa

**DOI:** 10.1002/mco2.70212

**Published:** 2025-06-06

**Authors:** Andrea Iannucci, Marco Colella, Macarena Quiroga, Rachele Frascatani, Lorenzo Tomassini, Claudia Maresca, Eleonora Franzè, Federica Laudisi, Giuseppe Sica, Irene Marafini, Alessandro Michienzi, Ivan Zanoni, Giovanni Monteleone, Ivan Monteleone

**Affiliations:** ^1^ Department of Biomedicine and Prevention University of Rome "Tor Vergata" Rome Italy; ^2^ Department of Systems Medicine University of Rome "Tor Vergata" Rome Italy; ^3^ Epithelial Plasticity and Metastasis Group Instituto de Investigación Biomédica de A Coruña (INIBIC) Complexo Hospitalario Universitario de A Coruña (CHUAC) Sergas Universidade da Coruña (UDC) A Coruna Spain; ^4^ Department of Surgery University of Rome "Tor Vergata" Rome Italy; ^5^ Azienda Ospedaliera Policlinico Tor Vergata Rome Italy; ^6^ Division of Immunology and Division of Gastroenterology Harvard Medical School and Boston Children's Hospital Boston Massachusetts USA

**Keywords:** adenosine deaminase acting on RNA 1, inflammatory bowel disease, innate immunity, mucosal damage, panoptosis

## Abstract

The gut virome is a complex community that exists in equilibrium with the host. Disruptions of this balance could drive the development of inflammatory diseases, such as inflammatory bowel disease (IBD). RNA editing, particularly A‐to‐I editing by ADAR1, prevents the excessive immune response to viral double strand (ds) RNA. Failure of RNA editing may sustain inflammation and this study explore the role of ADAR1 in IBD. ADAR1 was analyzed in IBD patients and healthy controls (CTR) using western blotting and qPCR. Colonic epithelial cells (HCEC‐1CT), ex vivo organ cultures, and colonic organoids were treated poly I:C after ADAR1 silencing with an antisense oligonucleotide (AS). Inflammatory pathways and PANoptosome were measured by western blotting, flow cytometry, and ELISA. The role of ADAR1 was also studied in DSS‐colitis model. ADAR1 was significantly reduced in the inflamed epithelium of ulcerative colitis (UC) gut samples. ADAR1 silencing in HCEC‐1CT, ex vivo organ cultures or colonic organoids strongly increases the immune response to poly I:C and leads to activation of inflammatory pathways and PANoptosis. Inhibition of gut ADAR1 expression during DSS‐colitis exacerbated gut inflammation. JAK inhibition or AhR activation mitigated the immune response that follows ADAR1 silencing. These data suggest that ADAR1 could be involved in IBD inflammation.

## Graphical abstract

The gut virome is a complex community that, when disrupted, may contribute to inflammatory diseases like IBD. We found that ADAR1 expression is reduced in gut inflamed mucosa of ulcerative colitis patients. ADAR1 silencing heightened activation of inflammatory pathways and PANoptosis exacerbating gut tissue damage, but JAK inhibition or AhR activation helped mitigate this immune response

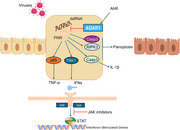

## 1 Introduction

Inflammatory bowel disease (IBD) represents a group of chronic inflammatory conditions of the gastrointestinal tract, primarily including Crohn's disease (CD) and ulcerative colitis (UC) [1, 2, 3]. These conditions are characterized by recurring episodes of inflammation that can lead to various gastrointestinal symptoms such as abdominal pain, diarrhea, and weight loss [2, 3].

The incidence of IBD has been increasing globally, particularly in regions undergoing rapid industrialization and urbanization, suggesting that environmental factors play a significant role in its pathogenesis [4]. Indeed, pathophysiology of IBD involves a complex interplay between genetic predisposition, immune response, and environmental triggers [1]. This harmful interaction drives a complex and multifaceted immune response, involving both the innate and adaptive gut immune systems [1]. In IBD, the innate immune response, which serves as the first line of defense against pathogens, is often dysregulated leading to an inappropriate activation of the immune system, causing chronic inflammation [1, 5, 6]. This is further exacerbated by the adaptive immune system, where T cells and other immune components produce cytokines and chemokines that contribute to the inflammatory process [5, 6]. The final result is that the balance between pro‐inflammatory and anti‐inflammatory signals is disrupted, leading to the tissue damage and clinical manifestations observed in IBD [7].

Among the various environmental factors that certainly influence the IBD inflammatory progression or expansion, intestinal dysbiosis has been recognized as pivotal for its evolution [8, 9]. Among several intestinal commensals, emerging data indicate that the viral component of the microbiome, termed virome, can profoundly influence host physiology [10, 11]. Viruses can interact with the host immune system in various ways, potentially altering immune responses and contributing to the chronic inflammation seen in IBD [11, 12, 13]. A pioneer study indicates that in IBD enteric virome is abnormal and is associated with a significant expansion of distinct species [11]. Importantly, expansion and diversification of the enteric virome was not secondary to changes in bacterial populations [11]. Moreover, a recent paper found that eukaryotic viruses, enriched from non‐IBD, noninflamed human colon resections, actively elicited peculiar anti‐inflammatory innate immune programs [12]. Conversely, IBD colon resection viromes provoked inflammation and consequently mucosal damage [12]. These data support a model in which changes in the virome may contribute to intestinal inflammation and gut dysbiosis. In this regard, the *Orthohepadnavirus* was detected in about 45% of patients with UC and found to prompt colitis in mice, while its suppression dampened inflammation in vivo [13].

Understanding the intricate relationship between the gut immune system and the virome is crucial for developing new therapeutic strategies aimed at modulating the immune response and improving outcomes for IBD patients. Recent findings have highlighted the role of RNA editing, an enzymatic process that drives the deamination of adenosines to inosines (A‐to‐I editing) by adenosine deaminase acting on RNA 1 (ADAR1), to control the immune reaction to viral as well as self double‐stranded RNA (dsRNA) [14]. In humans, ADAR1 is mutated in a rare autosomal‐dominant disease, such as Aicardi–Goutières syndrome (AGS), which affects its catalytic activity, leading to a total and uncontrolled activation of its downstream intracellular dsRNA sensor pathway [15, 16]. Furthermore, a recent study indicates that ADAR1 genetic variants are associated with common inflammatory diseases [17]. The authors found that the inflammatory disease risk variants were associated with reduced editing dsRNA and with increased interferon (IFN) responses, suggesting cellular dsRNA editing and sensing as a previously underappreciated mechanism of common inflammatory diseases. In animal models, ADAR1 is essential for intestinal stem cell maintenance by suppressing endoplasmic reticulum (ER) stress and IFN signaling [18, 19] while, in human celiac disease, a defective ADAR1 expression boosts the immune‐pathogenic responses to gliadin peptide [20]. In addition, ADAR1 has been recognized as a crucial negative regulator of PANoptosis, an inflammatory programmed cell death pathway regulated by the PANoptosome complex with key features of pyroptosis, apoptosis, and/or necroptosis that cannot be accounted for by any of these three pathways alone [21, 22]. However, whether and how intestinal ADAR1 acts in IBD remains an open question.

In this study, we identified a reduced expression of human ADAR1 in gut inflamed epithelial cells of UC patients. ADAR1 downmodulation drives an innate immune response and triggers gut inflammation. The intestinal dsRNA‐ADAR1‐mediated intracellular pathway could provide new insights into disease mechanisms and potential therapeutic targets.

## 2 Results

### 2.1 ADAR1 Expression Is Downregulated in the Gut Epithelial Cells of Ulcerative Colitis Patients

**FIGURE 1:**
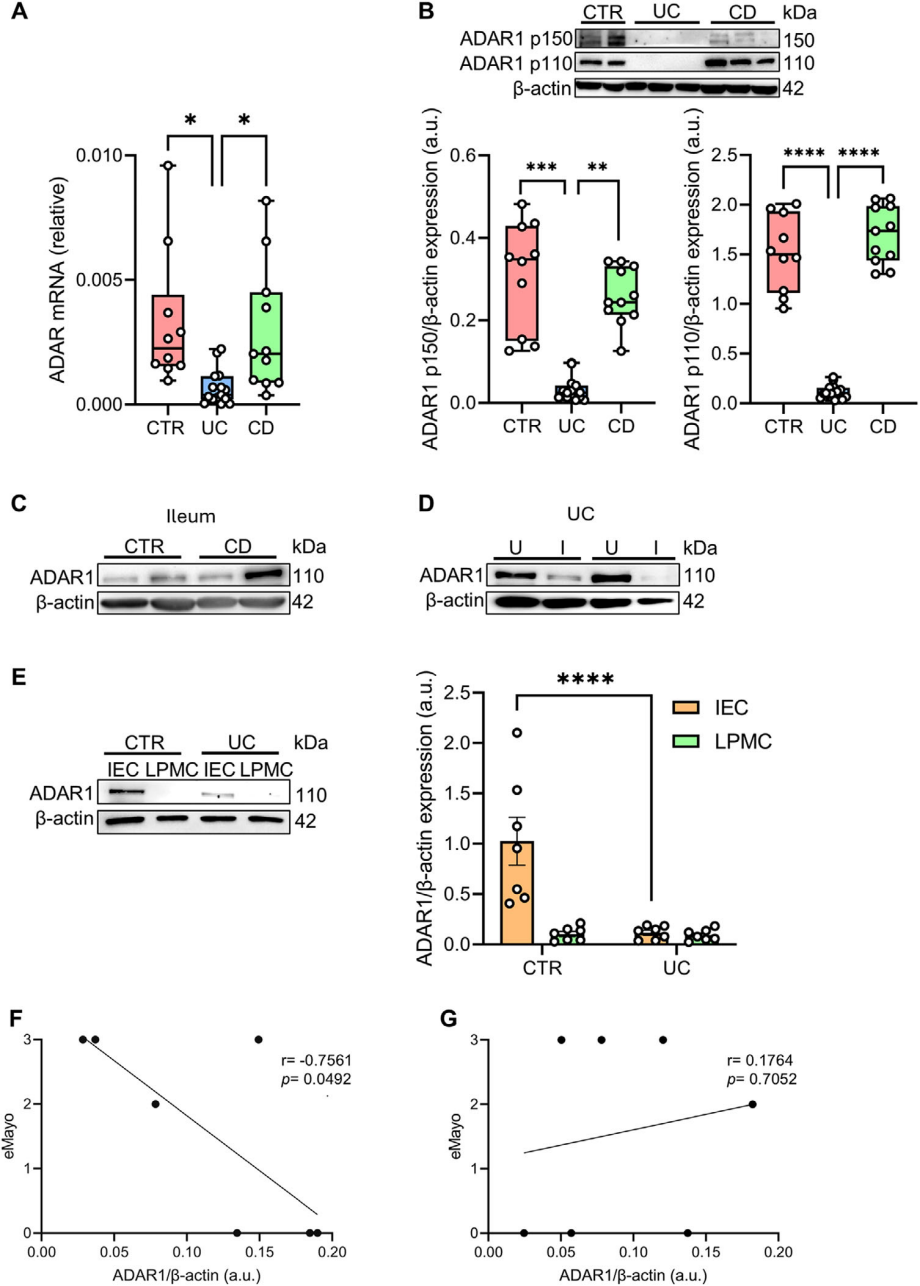
ADAR1 expression is reduced in inflamed gut of ulcerative colitis patients. (A) RNA expression of *ADAR* was evaluated by qRT‐PCR in colonic biopsies taken from 10 healthy controls (CTR), 15 patients with ulcerative colitis (UC), and 11 patients with Crohn's disease (CD). Values were normalized to β‐actin mRNA. Results are shown as box plots (25th to 75th percentile; horizontal lines represent median, maximum, and minimum values). *p < 0.05; one‐way ANOVA followed by Dunnett's test. (B) Representative western blot showing ADAR1 p150, ADAR1 p110, and β‐actin expression in tissue lysates from colonic biopsies of two CTR, three patients with UC, and three patients with CD. Lower panel shows the densitometric analysis of ADAR1/β‐actin ratio of the samples analyzed (left panel p150 isoform, right panel p110 isoform; 10 CTR, 15 patients with UC, and 11 patients with CD). Values are expressed as arbitrary units (a.u.). Results are shown as box plots (25th to 75th percentile; horizontal lines represent median, maximum, and minimum values). **p < 0.01, ***p < 0.001, ****p < 0.0001; one‐way ANOVA followed by Dunnett's test. (C) Representative western blot showing ADAR1 and β‐actin expression in tissue lysates from ileal biopsies of two CTR and two patients with CD. (D) Representative western blot showing ADAR1 and β‐actin expression in tissue lysates from paired colonic biopsies of uninflamed (U) and inflamed (I) areas of two patients with UC. (E) Representative western blot showing ADAR1 and β‐actin expression in intestinal epithelial cells (IEC) and lamina propria mononuclear cells (LPMC) in colonic biopsies of one CTR and one patient with UC. Right panel shows the densitometric analysis of ADAR1/β‐actin ratio of the samples analyzed (7 CTR and 7 patients with UC). Values are expressed in arbitrary units (a.u.). Data are shown as mean ± SEM. ****p < 0.0001; two‐way ANOVA followed by Sidak's test. (F) Correlation of densitometric analysis of ADAR1/β‐actin ratio in IEC of UC patients with their relative Mayo endoscopic score (eMayo). Each dot is a single biopsy. Data analyzed by Pearson's correlation. (G) Correlation of densitometric analysis of ADAR1/β‐actin ratio in LPMC of UC patients with their relative Mayo endoscopic score (eMayo). Each dot is a single biopsy. Data analyzed by Pearson's correlation.

Mucosal colonic biopsies taken from patients with CD or UC, along with healthy controls (CTR), were assessed for the expression of ADAR1 both at the mRNA and protein level. *ADAR*, mRNA, and ADAR1 protein expression (constitutive form, namely ADAR p110) were found to be significantly downregulated in colonic biopsies of patients with UC, compared to patients with CD or CTR, while no significant differences were observed between patients with CD and controls (CTR) (Figure 1A,B). Interestingly, a reduction of the inducible ADAR p150 isoform was also observed, supporting the idea that in UC all the ADAR1 isoforms are downregulated (Figure 1B). ADAR1 protein expression was also unaltered in ileal mucosal samples of CD patients compared to CTR (Figure 1C). Remarkably, western blot analyses in paired biopsy samples taken from inflamed and uninflamed areas of UC patients showed that ADAR1 protein expression was reduced in areas with active inflammation (Figure 1D). To further dissect whether ADAR1 downregulation in UC affected both the lamina propria and/or epithelial compartments, lamina propria mononuclear cells (LPMC) and intestinal epithelial cells (IEC) were isolated from colon tissue of UC patients or CTR. Strikingly, western blot analyses revealed a significant reduced ADAR1 protein expression in IEC of UC compared to CTR and such expression inversely correlated with Mayo endoscopic activity (Figure 1E,F). In contrast, in LPMC ADAR1 protein expression was unchanged between patients with UC and CTR, and no correlation was observed with Mayo endoscopic activity (Figure 1E,G). Taken together, these results show that ADAR1 is strongly downregulated in IEC of patients with UC, particularly in tissue exhibiting more severe inflammation.

### 2.2 Downregulation of ADAR1 in Human Gut Epithelium Enhanced dsRNA‐Mediated Inflammatory Responses

**FIGURE 2:**
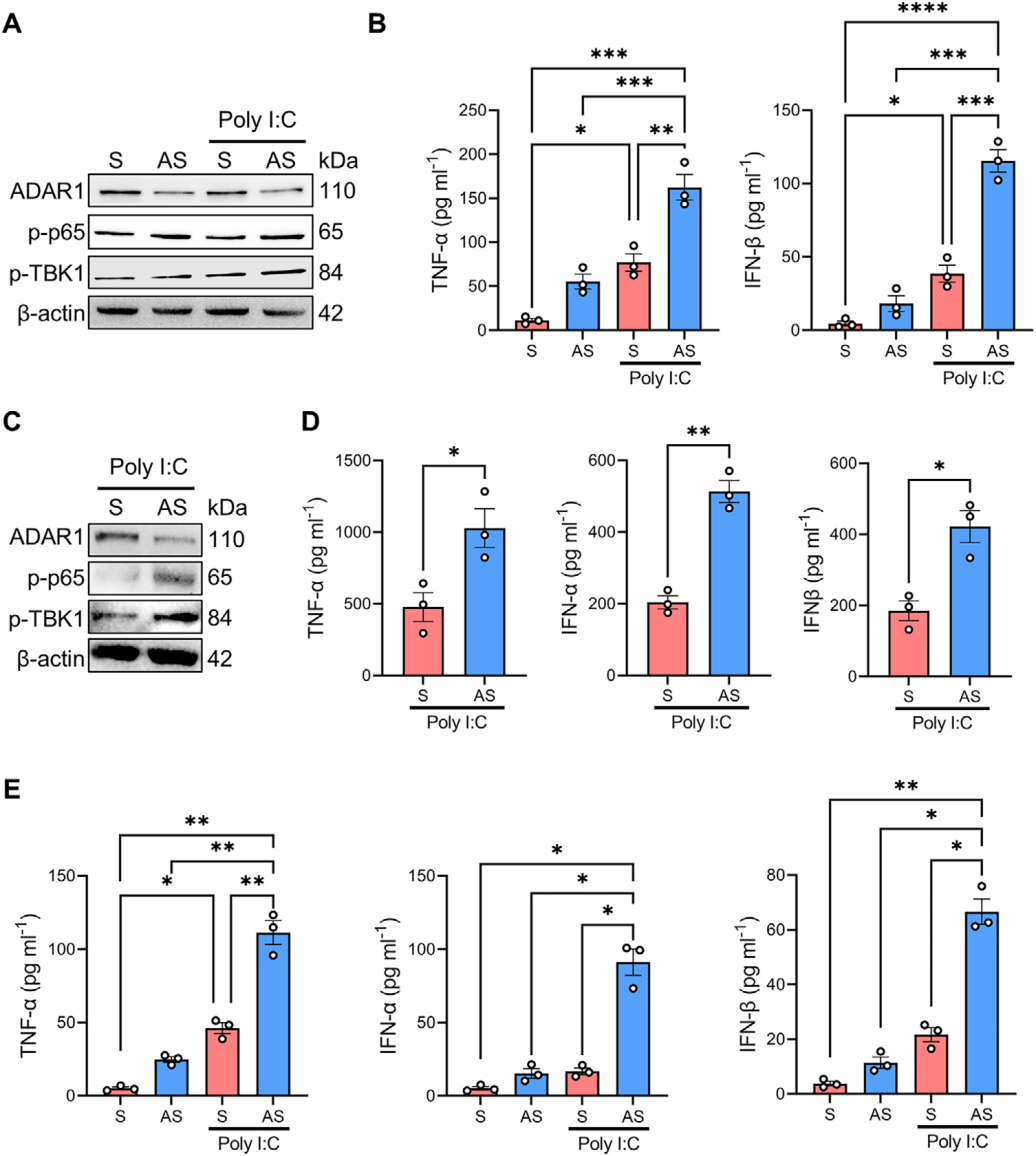
Knock‐down of ADAR1 increases gut epithelial inflammation. (A) Representative western blot showing ADAR1, active p65 (phosphorylated p65 or p‐p65), active TBK1 (phosphorylated TBK1 or p‐TBK1), and β‐actin expression in HCEC‐1CT transfected with ADAR1 sense (S) or antisense (AS) oligonucleotides for 24 h and then stimulated or not with poly I:C for 1 h. (B) Protein concentration of TNF‐α and IFN‐β determined by ELISA in the culture supernatants harvested from HCEC‐1CT transfected with ADAR1 S or AS for 24 h and then stimulated or not with poly I:C for 16 h. Data are expressed as mean ± SEM of three independent experiments (*p < 0.05, **p < 0.01, ***p < 0.001, ****p < 0.0001; two‐way ANOVA followed by Sidak's test). (C) Representative western blot showing ADAR1, active p65 (phosphorylated p65 or p‐p65), active TBK1 (phosphorylated TBK1 or p‐TBK1), and β‐actin expression in ex vivo organ cultures of healthy colonic mucosal samples transfected with ADAR1 S or AS for 20 h and then stimulated with poly I:C for 1 h. (D) Protein concentration of TNF‐α, IFN‐α, and IFN‐β determined by ELISA in the culture supernatants harvested from ex vivo organ cultures transfected with ADAR1 S or AS for 16 h and then stimulated with poly I:C for 8 h. Data are expressed as mean ± SEM of three independent experiments (*p < 0.05, **p < 0.01; Student's *t* test). (E) Protein concentration of TNF‐α, IFN‐α, and IFN‐β determined by ELISA in the culture supernatants harvested from colon organoids derived from colonic biopsies of healthy controls transfected with ADAR1 S or AS for 24 h and then stimulated or not with poly I:C for 16 h. Data are expressed as mean ± SEM of three independent experiments (*p < 0.05, **p < 0.01, ***p < 0.001, ****p < 0.0001; two‐way ANOVA followed by Sidak's test).

Given that both self and viral dsRNA are recognized by multiple pattern recognition receptors (PRRs), leading to the activation of inflammatory responses, we hypothesized that the loss of ADAR1 in IEC might influence the response to these stimuli. To test this, healthy intestinal epithelial cells (HCEC‐1CT) were incubated with a specific ADAR1 sense (S) or antisense (AS) oligonucleotide and then stimulated, or not, with poly I:C. Treatment of HCEC‐1CT with the ADAR1 AS, but not with S, reduced ADAR1 expression (Figure 2A). ADAR1 suppression was associated with increased phosphorylation of p65 and TBK1 in response to poly I:C, indicating a stronger activation of the intracellular inflammatory pathway response (Figure 2A). Consistent with this, ADAR1 AS‐treated HCEC‐1CT exhibited a significant increase in TNF‐α and IFN‐β release in cell culture supernatants in response to poly I:C, compared to S‐treated cells (Figure 2B). Similar results were obtained when using 3p‐hpRNA or 5′ppp‐dsRNA as dsRNA synthetic ligands (data not shown). To further investigate whether reduced ADAR1 expression could affect the inflammatory response in gut tissues, ex vivo organ cultures of healthy colonic mucosal samples were treated with ADAR1 S or AS oligonucleotides and then challenged with poly I:C. As observed in epithelial cells, ADAR1 AS‐treated organ cultures had a heightened inflammatory response to poly I:C, as evidenced by increased phosphorylation of p65 and TBK1 (Figure 2C), compared to S‐treated organ cultures. Likewise, ADAR1 knockdown was also accompanied by a significant increase in TNF‐α, IFN‐α, and IFN‐β release following poly I:C challenge (Figure 2D). Finally, to comprehensively assess the role of ADAR1 in modulating the inflammatory response of gut epithelium, we utilized intestinal organoids derived from colonic biopsies of healthy controls. ADAR1 AS‐treated organoids released significantly higher levels of TNF‐α, IFN‐α, and IFN‐β than their S‐treated counterparts following poly I:C challenging (Figure 2E). Collectively, these results demonstrate that reduced ADAR1 expression in IEC leads to an excessive inflammatory response to dsRNA.

### 2.3 ADAR1 Downregulation Compromises the Survival of Intestinal Epithelial Cells

**FIGURE 3:**
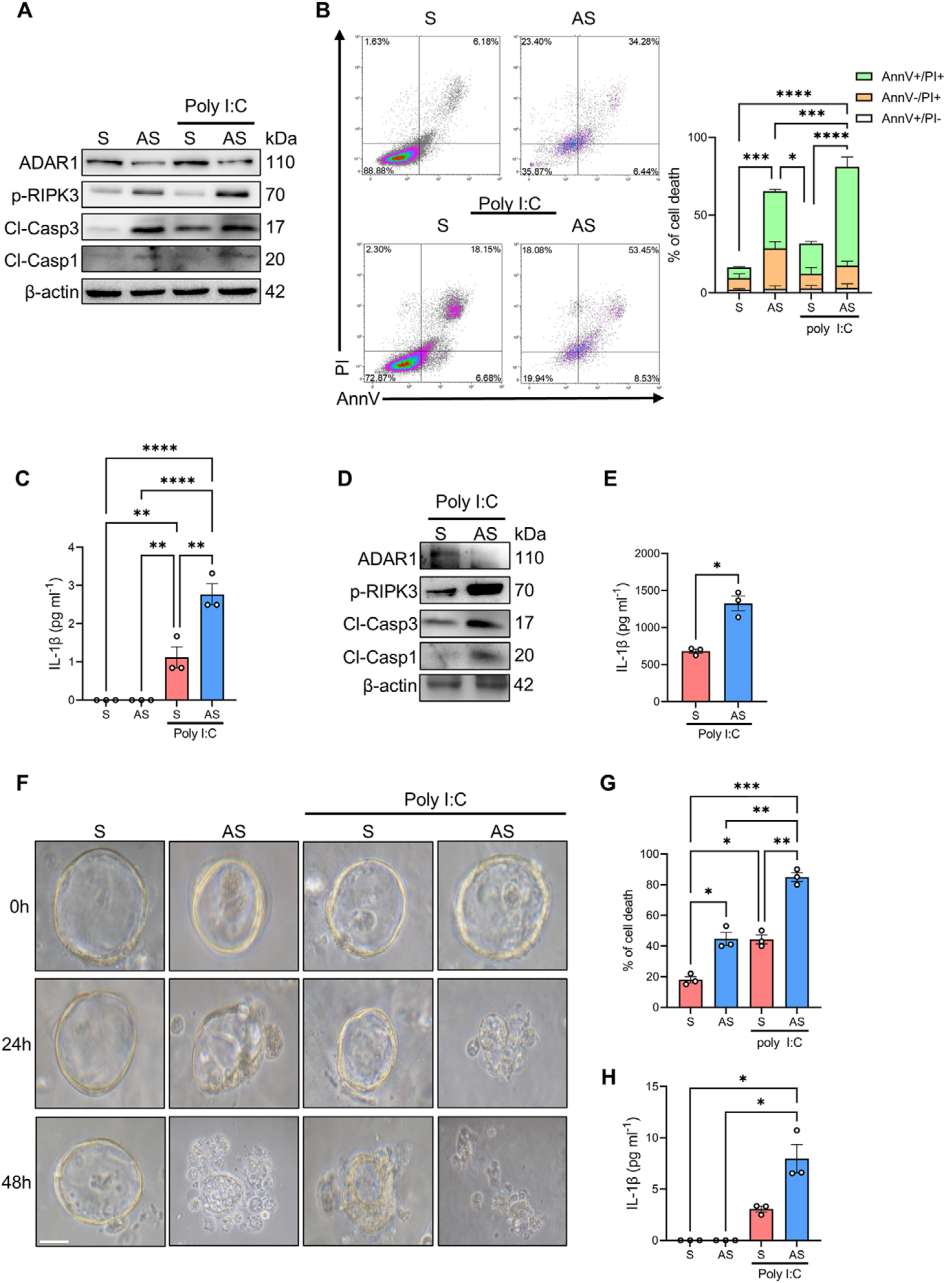
Knock‐down of ADAR1 induces PANoptosis. (A) Representative western blot showing ADAR1, active RIPK3 (phosphorylated RIPK3 or p‐RIPK3), active caspase 3 (cleaved caspase 3 or Cl‐Casp3), active caspase 1 (cleaved caspase 1 or Cl‐Casp1), and β‐actin expression in HCEC‐1CT transfected with ADAR1 sense (S) or antisense (AS) oligonucleotides for 24 h and then stimulated or not with poly I:C for 48 h. (B) Representative dot plot of annexin V (AnnV)‐ and propidium iodide (PI)‐positive HCEC‐1CT treated as indicated in (A). Right panel shows quantification of the percentage of AnnV and/or PI‐positive cells. Data are expressed as mean ± SEM of three independent experiments (the *p* value refers to comparison of AnnV+/PI+ cells among the groups; *p < 0.05, ***p < 0.001, ****p < 0.0001; two‐way ANOVA followed by Sidak's test). (C) Protein concentration of IL1‐β determined by ELISA in the culture supernatants harvested from HCEC‐1CT treated as in (A). Data are expressed as mean ± SEM of three independent experiments (**p < 0.01, ****p < 0.0001; two‐way ANOVA followed by Sidak's test). (D) Representative western blot showing ADAR1, active RIPK3 (phosphorylated RIPK3 or p‐RIPK3), active caspase 3 (cleaved caspase 3 or Cl‐Casp3), active caspase 1 (cleaved caspase 1 or Cl‐Casp1), and β‐actin expression in ex vivo organ cultures of healthy colonic mucosal samples transfected with ADAR1 sense (S) or antisense (AS) oligonucleotides for 16 h and then stimulated with poly I:C for 8 h. (E) Protein concentration of IL1‐β determined by ELISA in the culture supernatants harvested from ex vivo organ cultures treated as in (D). Data are expressed as mean ± SEM of three independent experiments (*p < 0.05, **p < 0.01; Student's *t* test). (F) Representative images of colon organoids derived from colonic biopsies of healthy controls transfected with ADAR1 sense (S) or antisense (AS) oligonucleotides for 24 h and then stimulated or not with poly I:C for 48 h. Three time points are shown after poly I:C challenging (0, 24, and 48 h). Scale bars: 100 µm. (G) Quantification of cell death (%) in colon organoids treated as in (F) measured by trypan blue dye. Data are expressed as mean ± SEM of three independent experiments (*p < 0.05, **p < 0.01, ***p < 0.001; two‐way ANOVA followed by Sidak's test). (H) Protein concentration of IL1‐β determined by ELISA in the culture supernatants harvested from colon organoids treated as in (F). Data are expressed as mean ± SEM of three independent experiments (*p < 0.05; two‐way ANOVA followed by Sidak's test).

ADAR1 has been recognized as a crucial negative regulator of PANoptosis, a highly inflammatory form of cell death that includes pyroptosis, apoptosis, and necrosis [21, 22]. Since an aberrant increase in the rate of IEC death is observed in several intestinal diseases such as UC [23], we sought to uncover whether defective ADAR1 expression in IEC could results in the activation of PANoptosome‐associated programmed cell death. To pursue this, ADAR1 AS‐ or S‐treated HCEC‐1CT were stimulated or not with poly I:C and then the primary mechanisms related to PANoptosis were analyzed, including the activation of caspase 3 (a marker of apoptosis), RIPK3 (a marker of necrosis), caspase 1, and the release of IL‐1β (markers of pyroptosis). ADAR1 knockdown in HCEC‐1CT resulted in the activation of RIPK3 (phosphorylated RIPK3), caspase 3 (cleaved caspase 3), and caspase 1 (cleaved caspase 1) along with increased cell death, as evidenced by AnnV/PI staining (Figure 3A,B). Moreover, poly I:C treatment further amplified IL1‐β release in ADAR1 AS‐treated HCEC‐1CT compared to S‐treated counterparts (Figure 3C). This pattern was mirrored also in ex vivo organ cultures of healthy colonic mucosa, where ADAR1 knockdown markedly enhanced poly I:C PANoptosome activation, leading to caspase 3, caspase 1, and RIPK3 activation and heightened IL‐1β release (Figure 3D,E). The impact of ADAR1 knockdown on PANoptosome‐associated programmed cell death was further examined in intestinal organoids derived from colonic biopsies of healthy controls. ADAR1 AS‐treated organoids exhibited swelling, disruption of the plasma membrane and cell death, while no morphological changes or cell death were observed in their S‐treated counterparts (Figure 3F,G). Furthermore, poly I:C treatment dramatically accelerated the loss of structure and increased cell death in ADAR1 AS‐treated organoids, alongside a significant boost in IL‐1β release compared to S‐treated counterparts (Figure 3F–H). Finally, mucosal colonic biopsies taken from patients with CD or UC along with CTR were assessed for the expression of PANoptotic markers. Noteworthy, a significant increase in the activation of RIPK3, caspase 3, and caspase 1 was found in the gut of UC patients compared to patients with CD or CTR (Figure S1). Overall, these findings further confirm the role of ADAR1 in preventing PANoptosis and suggest a mechanism by which reduced ADAR1 expression could contribute to the IEC death observed in UC.

### 2.4 ADAR1 Knockdown Worsens Gut Inflammation and Tissue Damage in DSS‐Induced Colitis

**FIGURE 4:**
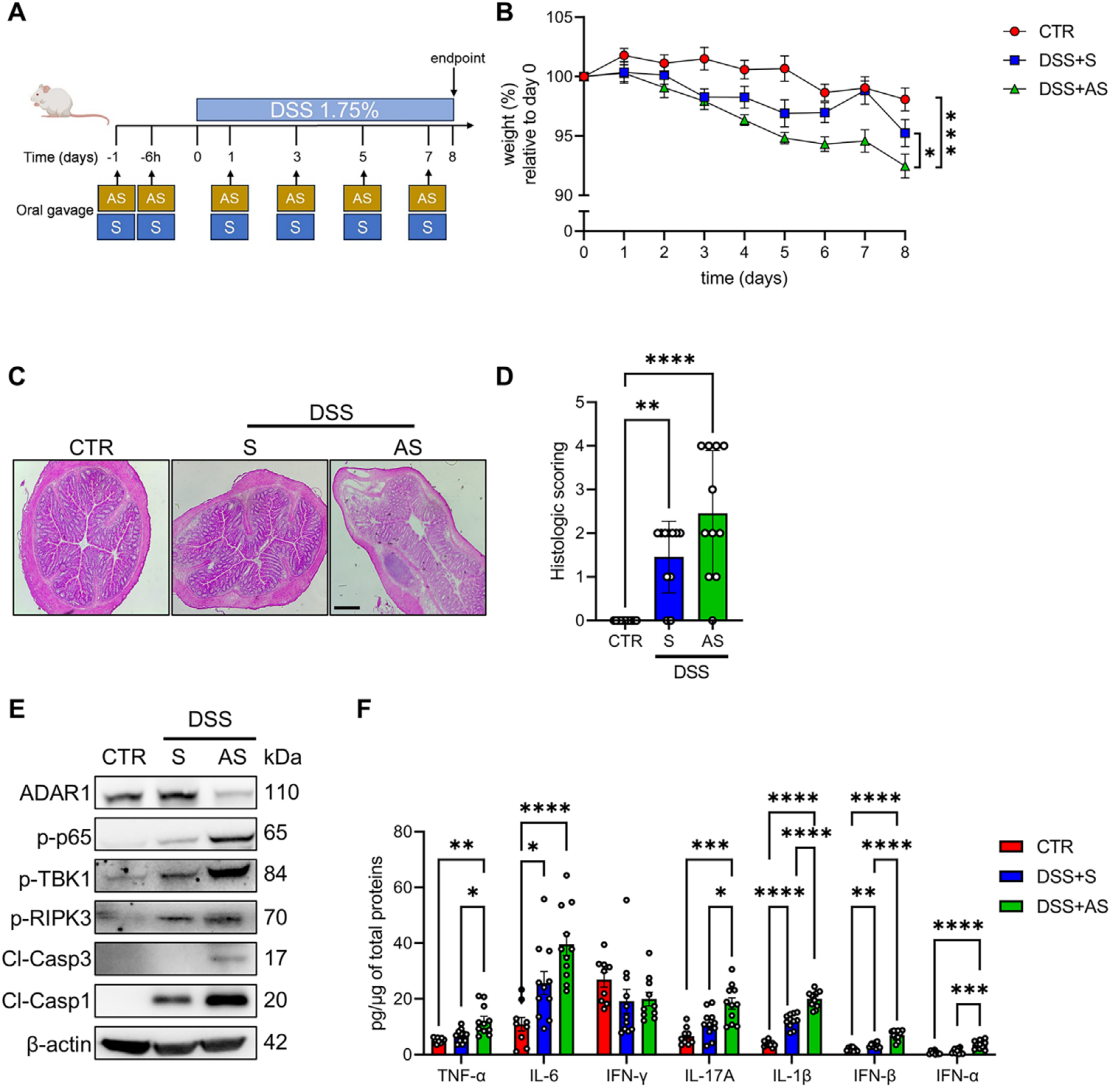
Mice with reduced expression of ADAR1 develop a more severe DSS‐induced colitis. (A) Scheme depicting DSS‐mediated colitis in which mice were given orally ADAR1 S or AS oligonucleotides 24 and 6 h before starting DSS administration (Day 0) and then every other day until Day 8. Control mice received regular drinking water (CTR). Two independent experiments were performed and at least 5 mice per group were used in each experiment. (B) Body weights of mice, from (A), were monitored daily and each point on the graph indicates the cumulative mean ± SEM of the body weights. (*p < 0.05, ***p < 0.001; two‐way ANOVA followed by Sidak's test). (C) Representative H&E‐stained colonic sections of mice from (A). Original magnification 20×. Right inset shows the histologic score of the colonic sections. Data are expressed as mean ± SEM. (**p < 0.01, ****p < 0.0001; one‐way ANOVA followed by Dunnett's test). (D) Representative western blot showing ADAR1, active p65, active TBK1, active RIPK3, active caspase 3, active caspase 1, and β‐actin expression of colonic tissue lysates of mice from (A). (E) Protein concentration of TNF‐α, IL‐6, IFN‐γ, IL‐17A, IL1‐β, IFN‐α, and IFN‐β determined by ELISA in the colonic tissue lysates of mice from (A). (*p < 0.05, **p < 0.01, ***p < 0.001, ****p < 0.0001; one‐way ANOVA followed by Dunnett's test).

We next investigated the role of ADAR1 in an experimental mouse model of dextran sulfate sodium (DSS)‐induced colitis. To this end, mice were given low‐dose DSS in drinking water for eight consecutive days, either with ADAR1 S or AS oligonucleotide treatment, or were left untreated (CTR) (Figure 4A). As expected, DSS administration in S‐treated mice caused body weight loss accompanied by a significant increase in tissue damage as confirmed by histological analysis and scoring of intestinal damage, compared to CTR mice (Figure 4B–D). Importantly, ADAR1 AS‐treated mice exhibited a greater reduction in body weights compared to CTR or S‐treated mice already at Day 3 or 5, respectively, upon DSS administration (Figure 4B). Furthermore, ADAR1 AS‐treated mice displayed markedly greater tissue damage compared to the other two groups (Figure 4C,D). To ascertain whether, as observed above in vitro and ex vivo, ADAR1 reduction correlates with heightened inflammation and PANoptosis also in vivo, we performed western blot and ELISA analyses in the colon lysates of the aforementioned mice. ADAR1 AS treatment led to a marked reduction of ADAR1 expression in colonic mucosa (Figure 4E). AS‐treated mice exhibited an increased activation of p65, TBK1, RIPK3, caspase 3, and caspase 1 alongside a significant production of proinflammatory cytokines (TNF‐α, IL‐6, IL‐17A, and IL‐1β) and IFNs (IFN‐α and IFN‐β) following DSS administration, compared to CTR and to their S‐treated counterparts (Figure 4E,F). Overall, these results underscore the protective role of ADAR1 in mitigating colitis, as its loss aggravates intestinal inflammation and tissue damage.

### 2.5 JAK Inhibition Attenuates *ADAR* Deficiency‐Induced Inflammation

**FIGURE 5:**
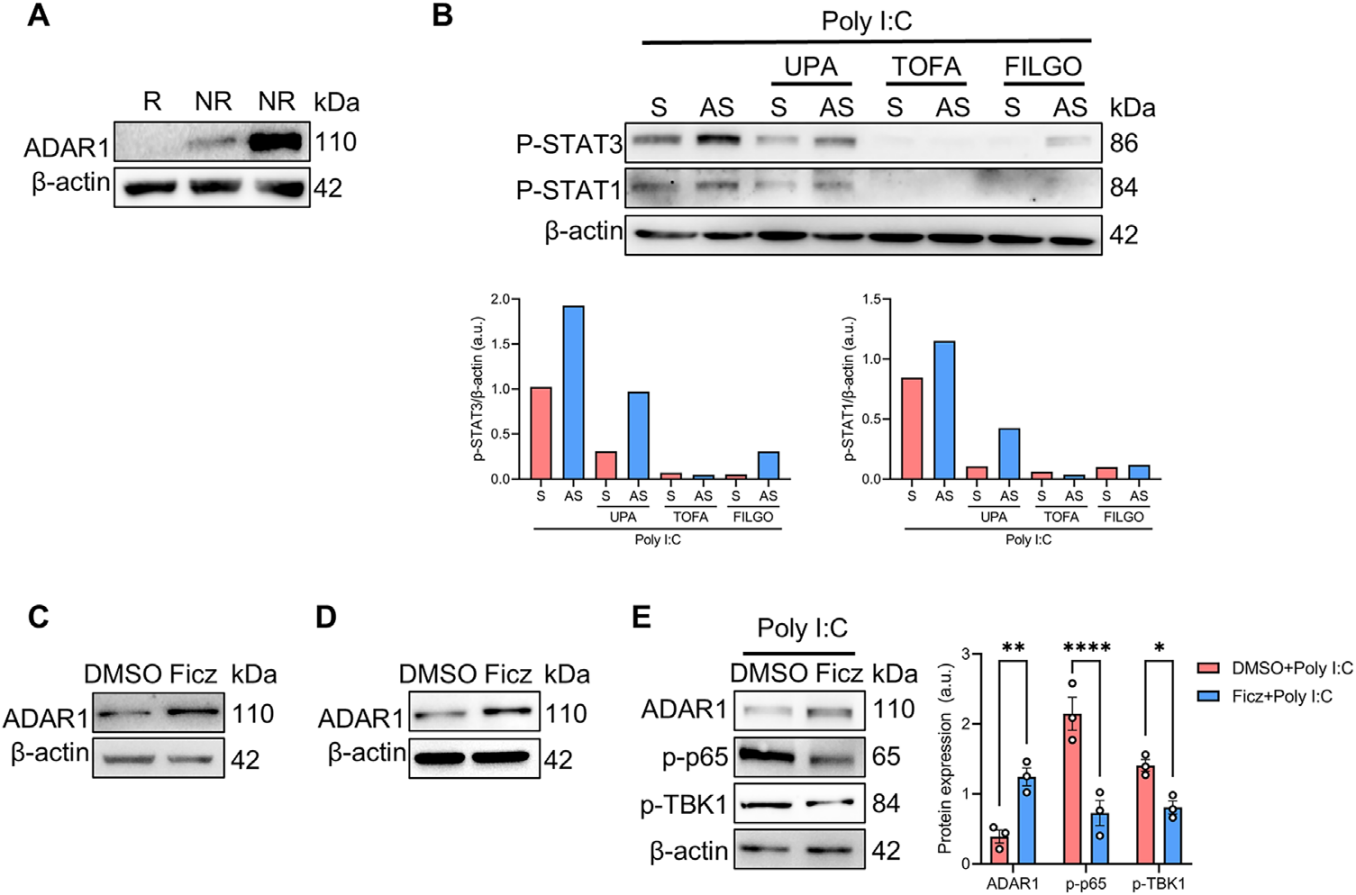
JAK inhibition or AhR activation prevent ADAR1 knock‐down mediated excessive immune responses to dsRNA. (A) Representative western blot showing ADAR1 and β‐actin expression in tissue lysates from colon biopsies of three patients with UC before JAKi treatment (R = responder, NR = non responder). (B) Representative western blot showing ADAR1, active STAT1 (phosphorylated STAT1 or p‐STAT1), active STAT3 (phosphorylated STAT3 or p‐STAT3), and β‐actin expression in ex vivo organ cultures of healthy colonic mucosal samples transfected with ADAR1 sense (S) or antisense (AS) oligonucleotides together or not with 1 µM of JAKis (upadacitinib or UPA, tofacitinib or TOFA, filgotinib or FILGO) for 20 h and then stimulated with poly I:C for 1 h. Right panels show the densitometric analysis of pSTAT1/β‐actin (upper panel) and pSTAT3/β‐actin (lower panel) ratio of the samples analyzed. Values are expressed as arbitrary units (a.u.). Data are representative of one experiment. (C, D) Representative western blot showing ADAR1 and β‐actin expression in HCEC‐1CT (C) or ex vivo organ cultures of UC patients' colonic mucosal samples (D) stimulated for 24 h with DMSO or Ficz (200 nM). (E) Representative western blot showing ADAR1, active p65 (phosphorylated p65 or p‐p65), active TBK1 (phosphorylated TBK1 or p‐TBK1), and β‐actin expression in ex vivo organ cultures of UC patients' colonic mucosal samples treated for 20 h with DMSO or Ficz (200 nM) and then stimulated with poly I:C for 1 h. Values are expressed as arbitrary units (a.u.). Right panel shows the densitometric analysis of ADAR/β‐actin, p65/ β‐actin, and pTBK1/β‐actin ratio of the samples analyzed. Values are expressed as arbitrary units (a.u.). Data are expressed as mean ± SEM of three independent experiments. (*p < 0.05, **p < 0.01, ****p < 0.0001; one‐way ANOVA followed by Dunnett's test).

IL‐6, IFN‐α, and IFN‐β, which are increased upon ADAR1 downregulation, activates the Janus kinase (JAK)‐signal transducer and activator of transcription (STAT). This prompted us to examine whether the inhibition of the JAK signaling pathway, which is emerging as a new treatment of IBD [24], might prove effective when ADAR1 expression is reduced. Initially, we analyzed ADAR1 protein levels in colonic biopsies of patients with UC just before they received a JAK inhibitor (JAKi) therapy. After 3 months of treatment, patients underwent colonoscopy and were divided into responders and nonresponders, based on their Mayo endoscopic activity score. ADAR1 expression in colonic biopsies of patients who responded to JAKi therapy was barely detectable, while higher expression was observed in colonic biopsies of nonresponder patients (Figure 5A). In following experiments, we wondered whether ADAR1 reduction could influence the efficacy of JAKis. Therefore, ex vivo organ cultures of healthy colonic mucosal samples were treated with ADAR1 AS or S along with three different JAKis (i.e., upadacitinib, tofacitinib, and filgotinib) and then stimulated with poly I:C. ADAR1 knockdown lead to increased phosphorylation of STAT1 and STAT3 after poly I:C challenging in ADAR1 AS‐treated organ cultures compared to their S‐treated counterparts (Figure 5B). However, when JAKis were applied, the phosphorylation of STAT1 and STAT3 was strongly reduced, becoming barely detectable when tofacitinib and filgotinib were employed. Altogether, these results suggest that, in the gut tissue, JAK inhibition may be effective in reducing excessive STAT activation driven by cytokines released in response to diminished ADAR1 expression.

### 2.6 The Aryl Hydrocarbon Receptor Induces ADAR1 Expression and Prevents dsRNA‐Mediated Immune Response in UC Patients

In an attempt to explore new factors capable of controlling ADAR1 expression, we took advantage of the JASPAR free web tool [25], which revealed several transcription factors (TFs) potentially binding to ADAR1 promoter (data not shown). Among these, we focused on the aryl hydrocarbon receptor (AhR) since its activation has been shown to ameliorate experimental colitis [26, 27]. In addition, data from the peak browser of ChIP‐Atlas [28, 29] showed that AhR is directly recruited to the promoter regions of *ADAR* in HepG2 and MCF‐7 cells. To confirm whether AhR induced the expression of ADAR1 in intestinal cells, HCEC‐1CT were treated with the AhR ligand Ficz (or with DMSO as a control). Activation of AhR was sufficient to increase the expression of ADAR1 (Figure 5C) and similar results were observed in ex vivo organ cultures of healthy colonic mucosal samples stimulated with Ficz (Figure 5D). Finally, to assess whether restoring ADAR1 expression could mitigate dsRNA‐induced inflammation in gut tissue, ex vivo organ cultures of UC patients' colonic mucosal samples were treated with Ficz or DMSO and then stimulated with poly I:C (Figure 5E). As expected, low ADAR1 expression led to increased phosphorylation of p65 and TBK1 in response to poly I:C. However, Ficz‐induced upregulation of ADAR1 resulted in a diminished inflammatory response to poly I:C, as evidenced by phosphorylation of p65 and TBK1. Collectively, these data show that AhR activation can enhance ADAR1 expression, thereby dampening dsRNA‐mediated inflammatory responses in the gut mucosa.

## 3 Discussion

The findings of this study provide significant insights into the role of ADAR1 in the pathophysiology of IBD, particularly UC. Our results reveal that ADAR1 expression is markedly downregulated in UC intestinal epithelium compared to CD and healthy controls. Noteworthy, this reduced expression correlates with higher endoscopic scores, suggesting that ADAR1 may serve as a potential biomarker for disease severity in UC. These observations are consistent with prior studies that have highlighted the differential expression of RNA‐editing enzymes in various immune mediated diseases [30, 31, 32], emphasizing the specific role of ADAR1 in modulating also intestinal inflammation. The limited human biological material available prevented us from assessing global editing in the gut tissue of UC patients and controls, however, it has been widely confirmed that a strong reduction of ADAR1 expression significantly impairs the physiological editing capacity present in tissue [14, 33]. So far, the molecular mechanism that drives the ADAR1 downregulation in UC gut epithelial cells remained elusive. Our data revealed that AhR agonist treatment is associated with increased ADAR1 expression thus suggesting a direct link between AhR activity and ADAR1. Several works have shown that in IBD intestinal mucosa there is reduced AhR expression or activity suggesting that the reduced expression of ADAR1 may be at least partly explained by a decreased AhR activity [26, 27, 34]. Nevertheless, we do not exclude the possibility that ADAR1 expression can be also controlled by other molecules/mechanism. Indeed, several studies have previously shown that the expression of ADAR1 is modulated by cytokines or miRNAs which are produced in different immune‐mediated diseases similar to UC [20, 31, 35], so the ADAR1 levels in UC mucosa can be controlled by an intricate interplay between these different molecules.

Our results contrast with a previous paper showing that ADAR1 is upregulated in UC mucosa [36], however, there are important differences to point out. First, the authors analyzed ADAR1 exclusively in UC rectal mucosa and not in other inflamed colon areas and more importantly, the high expression of ADAR1 was mainly present in patients with increased risk for carcinogenesis. In our study, we excluded patients who presented cancerous or precancerous lesions, and it is also important to point out that even in our population there are differences in ADAR1 expression between the various UC patients. Therefore, we do not exclude that patients with a greater expression of ADAR1 may be at increased risk to develop colon cancer in the near future.

The experiments conducted on human intestinal epithelial cell lines, organoid cultures, and organoids derived from colon biopsies of healthy individuals, stimulated with a synthetic analog of dsRNA after ADAR1 downregulation, further elucidate the functional consequences of reduced ADAR1 expression. The induction of a robust inflammatory response characterized by increased production of pro‐inflammatory cytokines, such as Type I IFN and TNF‐α, underscores the importance of ADAR1 in maintaining epithelial homeostasis. This is in line with previous research suggesting that epithelial ADAR1 expression modulates the inflammatory response and intestinal homeostasis, through its editing activity on dsRNA [18, 19]. Moreover, the observation that downregulation of ADAR1 leads to enhanced activation of PANoptosis leading to augmented necroptosis, apoptosis and IL‐1β release, provides novel insights into the mechanistic pathways by which ADAR1 influences UC gut inflammation. The activation of these pathways, in the absence of sufficient ADAR1 activity, suggests that ADAR1 may play a protective role in preventing excessive epithelial cell death, which could contribute to the chronicity and severity of inflammation observed in UC. Our results are in agreement with recent observations indicating that several key genes of PANoptosome are highly expressed in UC mucosa and correlated with an increase in pro‐inflammatory [37, 38]. Moreover, while our study was ongoing, two recent papers showed that depletion of ADAR1 resulted in Z‐RNA accumulation and activation of the Z‐RNA sensor ZBP1, which culminated in PANoptosome activation [39, 40]. Further studies are needed to understand whether ADAR1‐dependent PANoptosis in UC is also due to an activation of ZBP1, but, in line with the recent studies mentioned above, we also confirmed that ADAR1 downregulation drives the activation of the PANoptosome.

Our in vivo findings in the DSS‐induced colitis model further corroborate the in vitro data. Mice with reduced ADAR1 expression exhibited a greater susceptibility to severe colitis, with increased activation of inflammatory pathways, cytokine release, and cell death pathways. The exacerbated disease observed in these mice parallels the clinical findings in UC patients with low ADAR1 expression, suggesting that restoring ADAR1 levels could mitigate disease progression. Furthermore, a recent study has shown that ADAR1‐mediated RNA editing contributes to prevent inflammatory responses in small intestine mucosa and, moreover, inflammatory gastrointestinal disorders are sometimes observed in patients with ADAR1 gene mutations [19, 41]. It is plausible that gut‐derived virome dsRNA could contribute to enhanced colitis in conditions of reduced ADAR1 expression, as ADAR1 activity helps to prevent innate immune activation against dsRNA. Indeed, it has already been demonstrated that synthetic dsRNA synthetic analogs (poly I:C) significantly enhanced inflammation in mice model of small intestinal damage with downregulated ADAR1 [20]. Future studies will be directed to discriminate the role of endogenous dsRNA from viral or other origin dsRNA in this context. Since *ADAR* is expressed in different cell types, the use of oral AS oligonucleotide specific for ADAR1 in mice cannot rule out which cells are responsible for the exacerbation of the inflammation, although a previous study showed that in vivo administration of AS DNA can be used to manly target epithelial cells, minimizing systemic AS distribution and its potential secondary effects [42].

Our study also highlights the potential therapeutic implications of our findings. The responsiveness of UC patients with low ADAR1 expression to JAK inhibitors, along with the reduced activation of STAT1 and STAT3 in organ cultures treated with JAK inhibitors following ADAR1 downregulation, suggests a possible interplay between ADAR1‐dsRNA and the JAK‐STAT signaling pathway. Indeed, a previous study has shown that DNA sensor‐associated Type I interferon signaling is increased in UC patients and this inflammatory response was blocked by the JAK inhibitor [43]. Several cytokines, including IFNs, IL‐6, IL‐12, IL‐23, IL‐21, and IL‐13, have been shown to act as mediators of pathological responses in UC patients [5] and many of these cytokines converge on the JAK‐STAT signaling pathway, including JAK1, JAK2, JAK3, and tyrosine kinase 2 (TYK2), that have been explored as therapeutic targets in IBD [24]. Future studies should aim to validate these findings in larger cohorts of UC patients, but our findings suggest that inhibition of the JAK pathway is an effective approach to mitigate ADAR1 deficiency‐induced bowel inflammation. This finding is particularly relevant given the emerging role of JAK inhibitors in the treatment of UC, indicating that ADAR1 expression levels might predict therapeutic response. Our findings also open the possibility of combining JAK inhibitors with strategies aimed at modulating ADAR1 activity to enhance therapeutic efficacy.

In conclusion, this study highlights the critical role of ADAR1 in the regulation of intestinal inflammation and its potential as a biomarker and therapeutic target in UC. Our data support that the centrality of the ADAR1‐dsRNA‐JAK/STAT pathway during UC development offering new opportunities for clinical interventions.

## 4 Materials and Methods

### 4.1 Patients and Human Samples

Mucosal biopsies were taken from the inflamed areas of 25 patients with UC (median age 45.5 years, range 17–76 years, 56%/44% F/M), 11 patients with colonic CD (median age 44 years, range 18–68 years, 45%/55% F/M), and 4 patients with ileal CD undergoing colonoscopy (median age 57 years, range 37–73 years, 50%/50% F/M). Moreover, paired biopsies were taken from the inflamed and uninflamed mucosa of two additional UC patients. Endoscopic activity was assessed using the Mayo endoscopic score in UC patients [44]. At the time of biopsy sampling, 1 out of 25 patients with UC and 3 out of 15 patients with CD were receiving no therapy, 15 with UC and 8 with CD mesalamine, 1 with UC thiopurines, 2 with UC corticosteroids, 3 with UC (1 infliximab, 1 vedolizumab, and 1 adalinumab), 4 with CD (2 infliximab and 2 adalinumab) biologics, and 3 with UC JAK inhibitors (2 upadacitinib and 1 filgotinib). Healthy controls included biopsies taken from macroscopically and microscopically unaffected colonic and ileal mucosa of 21 subjects undergoing colonoscopy for colon cancer screening (median age 54 years, range 40–64, 57/43% F/M). All colonoscopy procedures were performed at the Gastrointestinal Unit of Policlinico di Tor Vergata (Rome, Italy). Informed consent was obtained from all the patients.

### 4.2 Cell Isolation and Cultures

Human IEC and LPMC were isolated from biopsies of UC patients or healthy controls [26]. Briefly, after mucus elimination using dithiothreitol (DTT Cat #D0632; Sigma–Aldrich, Milan, Italy), biopsies were incubated with ethylenediaminetetraacetic acid (EDTA Cat #E7889; Sigma–Aldrich) and the detached epithelial cells collected. After that, biopsies were digested with liberase‐tm and DNase I (both 0.2 mg/mL; Cat #11284932001 and #633131, respectively; Roche, Basel, Switzerland) and LPMC collected. CD3 protein expression was used to confirm that no immune cells contamination occurred when collecting epithelial cells.

The human normal colonic epithelial cell line HCEC‐1CT was obtained from EVERCYTE GmbH (Vienna, Austria) and cultured in ColoUp medium (Cat #MHT‐039, EVERCYTE GmbH). Cells were transfected with ADAR1 AS or S oligonucleotides (IDT, Coralville, IA, USA; 100 nM) [20] for 24 h using Opti‐MEM medium and Lipofectamine 3000 according to the manufacturer's instructions (Thermo Fisher Scientific, Waltham, MA, USA) and then incubated with poly I:C alone or complexed with Lyovec (5 µg/mL, Cat #TLRL‐PIC Invivogen, San Diego, CA, USA). In addition, cells were treated for 24 h with DMSO or Ficz (200 nM, Cat #MEDHY12451, MedChemExpress, Monmouth Junction, NJ, USA).

### 4.3 Ex Vivo Organ Cultures

Colonic mucosal samples were taken from macroscopically and microscopically unaffected colonic areas of patients undergoing surgery for colon cancer or from UC patients undergoing surgery for a chronic active disease unresponsive to medical treatment, transfected with AS or S oligonucleotides and cultured as previously described [45], alone or with upadacitinib, tofacitinib, and filgotinib (1 µM, Cat #HY19596, #HY‐40354, and #HY‐18300, respectively, MedChemExpress). After 16 h, organ cultures were treated with poly I:C alone or complexed with Lyovec (5 µg/mL). In addition, colonic mucosal samples from UC patients were treated for 20 h with DMSO or Ficz (200 nM), and then incubated with poly I:C (5 µg/mL).

### 4.4 Organoids Cultures and Treatments

Human organoids were generated from colonic biopsies taken from healthy controls. After washes with ice‐cold PBS, biopsies were thoroughly minced using sterile scissors, and then incubated with Gentle Cell Dissociation Reagent (Cat #100‐0485, Stemcell Technologies, Cambridge, UK). Isolated crypts were then passed through a 70 µm cell strainer (Corning, Sigma–Aldrich, Milan, Italy) and cultured using Corning Matrigel Matrix, Growth Factor Reduced, Phenol Red‐Free (Cat #356231, Corning) in the presence of IntestiCult Organoid Growth Medium (Cat #100‐0190, Stemcell Technologies), with the addition of antibiotics (penicillin/streptomycin Cat #PS‐B, and gentamycin #GEN‐10B, all from Capricorn Scientific, Ebsdorfergrund, Germany) and ROCKi (Cat #Y‐27632, Stemcell Technologies).

Established colon organoids were used for transfection and treatments. Before the transfection, colon organoids were dissociated using TrypLE Express (Cat #12604‐013, Thermo Fisher Scientific). Then, cells were plated in a single well of a 24‐well plate with 450 µL of IntestiCult Organoid Growth Medium with the addition of ROCKi and 50 µL of transfection mixture (3 µL of Lipofetamine 3000 and 200 nM of S or AS oligonucleotides in Opti‐MEM medium). The plate was then centrifuged at 32°C at 600 g for 1 h, followed by incubation at 37°C for 3 h. Cells were then harvested, pelleted, and cultured as described above. After 24 h transfected organoids were incubated with poly I:C (10 µg/mL) and cultured for 24 h to collect supernatants for cytokine release analysis or for 48 h to evaluate organoids morphology and cell death.

Organoids morphology was evaluated at 0, 24, and 48 h after poly I:C treatment using a Zeiss Axiovert 40 CFL inverted microscope (Carl Zeiss, Milan, Italy). Organoids cell death was measured after 48 h after poly I:C treatment using a hemocytometer. Briefly, organoids were dissociated using a previously described protocol to generate single cells and then mixed with trypan blue to distinguish live and dead cells [46].

### 4.5 Western Blotting

Total proteins were extracted in RIPA buffer using a TissueLyser II (Qiagen, Milan, Italy), or incubated for 30 min at 4°C in rotation, from tissues or cells, respectively, as indicated before [47]. Cell debris and membranes were cleared by centrifugation (30 min at 15,000 rpm) and then lysates separated by electrophoresis using SDS‐polyacrylamide gels and transferred to nitrocellulose membranes. After blocking, membranes were probed with specific primary and HRP‐conjugated secondary antibodies and proteins detected using SuperSignal West Dura Extended Duration Substrate (ThermoFisher Scientific), or Amersham ECL Detection Reagents (Cytiva, Marlborough, MA, USA) and images acquired using Quantity One software (BioRad, Hercules, CA, USA). The following primary antibodies were used: rabbit anti‐ADAR1 (Cat #81284), rabbit anti‐phospho‐NF‐κB p65 (Cat #3033), rabbit anti‐phospho‐TBK1 (Cat #5483), rabbit anti‐cleaved caspase 3 (Cat #9661), rabbit anti cleaved caspase 1 (Cat #89332), rabbit anti cleaved caspase 1 (Cat #4199), rabbit anti‐phospho‐STAT1 (Cat #9167), rabbit anti‐phospho‐STAT3 (Cat #9145) (all from Cell Signaling Technologies, Danvers, MA, USA, final concentration 1:1000), and rabbit anti‐phospho‐RIP3 (Cat #ab56164, Abcam, Cambridge, UK, final concentration 1:1000). Protein loading was normalized using mouse anti‐β‐actin (Cat #A1978, Sigma–Aldrich, final concentration 1:5000).

### 4.6 Cytokines Measurements

Cell‐cleared supernatants or mouse tissue lysates were analyzed for cytokine production using the following kits according to the manufacturer's instructions: human TNF‐α Quantikine ELISA Kit (Cat #DTA00C), human IL‐1 beta/IL‐1F2 Quantikine ELISA Kit (Cat #DLB50), mouse TNF‐α Quantikine ELISA Kit (Cat #MTA00B), mouse IL‐1 beta/IL‐1F2 Quantikine ELISA Kit ((Cat #MLB00C), mouse IL‐17 Quantikine ELISA Kit (Cat #M1700), mouse IL‐6 Quantikine ELISA Kit (Cat #M6000B), mouse IFN‐gamma Quantikine ELISA Kit (Cat # MIF00) (all from R&D systems, Minneapolis, MN, USA), VeriKine human IFN‐α ELISA Kit (Cat #41100), VeriKine human IFN‐β ELISA Kit (Cat # 41410), VeriKine mouse IFN‐α ELISA Kit (Cat #42120), and VeriKine mouse IFN‐β ELISA Kit (Cat #42400) (all from PBL assay science, Piscataway, NJ, USA).

### 4.7 RNA Extraction and Gene Expression Analysis

RNA was extracted using PureLink RNA Mini Kit (Cat #12183025, ThermoFisher Scientific) and then retrotranscribed using M‐MLV Reverse Transcriptase (Cat # 28025013, Thermo Fisher Scientific). The resulting complimentary DNA (cDNA) was analyzed for gene expression using iTaq Universal SYBR Green Supermix (Cat #1725120, BioRad) on a CFX Duet Real‐Time PCR System (BioRad). The following primers were used: human ADAR1 (forward: 5′‐GCTTGGGAACAGGGAATCG‐3′, reverse: 5′‐CTGTAGAGAAACCTGATGAAGCC‐3′), human β‐actin (forward: 5′‐AAGATGACCCAGATCATGTTTGAGACC‐3′, reverse: 5′‐AGCCAGGTCCAGACGCAGGAT‐3′). Gene expression variations were calculated using the ΔCT method.

### 4.8 Flow Cytometry

Flow cytometry was performed as previously described [48]. Briefly, after treatment, cells were collected, washed in annexin V (AnnV) buffer, stained with FITC‐AnnV (final dilution: 1:100; Cat#31490013, Immunotools, Friesoyte, Germany) and with 5 mg/mL of propidium iodide (PI) for 30 min at room temperature. Cells were then analyzed using Gallios flow cytometer (Beckman Coulter, Indianapolis, IN). AnnV‐/PI‐cells were considered as viable cells.

### 4.9 DSS‐Mediated Colitis

To induce colitis, 8 weeks‐old female Balb/c mice (Charles River Italia Srl, Lecco, Italy), were given 1.75% (w/v) DSS (Cat #160110, MP Biomedicals, Santa Ana, CA) in the drinking water for 8 days and then sacrificed through cervical dislocation. Twenty‐four and 6 h before starting DSS treatment, mice were given orally S or AS oligonucleotides (125 µg/mouse) as previously shown [20]. S and AS oligonucleotides were purchased by IDT and had the following sequences: 5′‐CCACAGATGACATCCCAGAT‐3 for S, and 5′‐ATCTGGGATGTCATCTGTGG‐3′ for AS. S or AS oligonucleotides administration proceeded every other day throughout the entire DSS treatment (8 days). A group of mice used as control was not given S or AS oligonucleotides and received regular drinking water. Body weight was monitored daily. Weight change was calculated as percentage of initial weight. The colons of the mice were collected for histology and total protein extraction. Cryosections of mouse colon samples were stained with hematoxylin and eosin, and the histologic scoring was performed in a blinded fashion as previously described [42].

### 4.10 Statistical Analysis

Statistical differences for comparison between groups were calculated using a two‐tailed Student's *t* test, or using one‐ or two‐way ANOVA for multiple comparison, and considered significant at a *p* value < 0.05. Statistical analyses were performed using GraphPad Prism version 9.0.0 for Windows (GraphPad Software, La Jolla, CA, USA, www.graphpad.com).

## Author Contributions

A.I. and I.Mo. designed the overall experiments and had unrestricted access to all data. A.I., M.C., R.F., E.F., F.L., and I.Mo. developed the methodology and protocols. A.I., M.C., M.Q., R.F., L.T., C.M., E.F. and F.L. performed experiments and/or data analyses. A.I. performed statistical analysis. G.S. and I.Ma. provided human samples. A.I. and I.Mo. wrote the first draft of the paper. A.I., A.M., I.Z., G.M., and I.Mo. reviewed and edited the manuscript. I.Mo. conceived and supervised the project. All authors read and approved the final article and take responsibility for its content.

## Ethics Statement

Each patient who enrolled in the study gave informed consent and the local Ethics Committee of the University hospital of Tor Vergata have approved the study protocol (n° 7.23 CET2 ptv). All the in vivo experiments were approved by the animal ethics committee (n° 699/2023‐PR) according to Italian legislation on animal experiments.

## Conflicts of Interest

The authors declare no conflicts of interest.

## Supporting information



Supporting Information

## Data Availability

Data used in this study are available from the corresponding author upon request.
