# Supplementary material for: Loss of Adenosine Deaminase Acting on RNA 1 Induces Panoptosis and Immune Response in Ulcerative Colitis Gut Mucosa
Source: MedComm (2020). 2025 Jun 6;6(6):e70212. doi: 10.1002/mco2.70212 (PMC12141924; doi:10.1002/mco2.70212)
Supplement: Supplementary file 1 — Supporting Information [file MCO2-6-e70212-s001.docx]

**LOSS OF ADENOSINE DEAMINASE ACTING ON RNA 1 INDUCES PANOPTOSIS AND IMMUNE RESPONSE IN ULCERATIVE COLITIS GUT MUCOSA**

Andrea Iannucci^1^, Marco Colella^2^, Macarena Quiroga^3^, Rachele Frascatani^2^, Lorenzo Tomassini^2^, Claudia Maresca^2^, Eleonora Franzè^2^, Federica Laudisi^2^, Giuseppe Sica^4^, Irene Marafini^5^, Alessandro Michienzi^1^, Ivan Zanoni^6^, Giovanni Monteleone^2,5^, Ivan Monteleone^1^

*^1^Department of Biomedicine and Prevention, University of Rome “Tor Vergata”, Rome, Italy*

*^2^Department of Systems Medicine, University of Rome “Tor Vergata”, Rome, Italy*

*^3^Epithelial Plasticity and Metastasis Group, Instituto de Investigación Biomédica de A Coruña (INIBIC), Complexo Hospitalario Universitario de A Coruña (CHUAC), Sergas, Universidade da Coruña (UDC), 15006 A Coruña, Spain*

*^4^Department of Surgery, University of Rome “Tor Vergata”, Rome, Italy*

*^5^Azienda Ospedaliera Policlinico Tor Vergata, 00133 Rome, Italy*

*^6^Division of Immunology and Division of Gastroenterology, Harvard Medical School and Boston Children's Hospital, Boston, MA, USA.*

**Address correspondence to**: Ivan Monteleone, MD, PhD, Department of Biomedicine and Prevention, University of Rome “Tor Vergata”, Via Montpellier 1, 00133 Rome, Italy.

E-mail: [ivan.monteleone@uniroma2.it](mailto:ivan.monteleone@uniroma2.it)


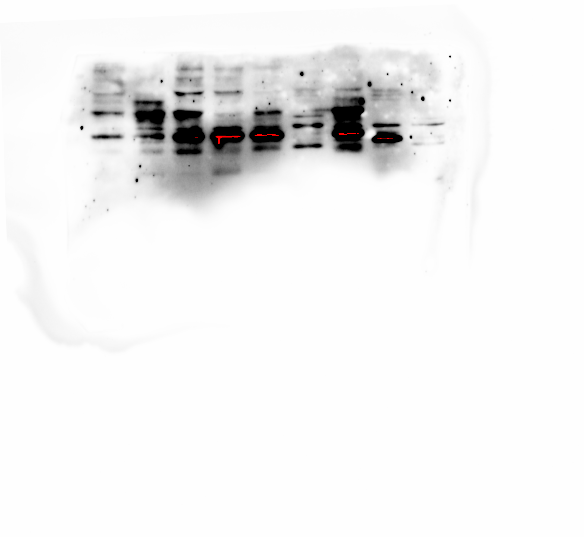


CTR

UC

CD


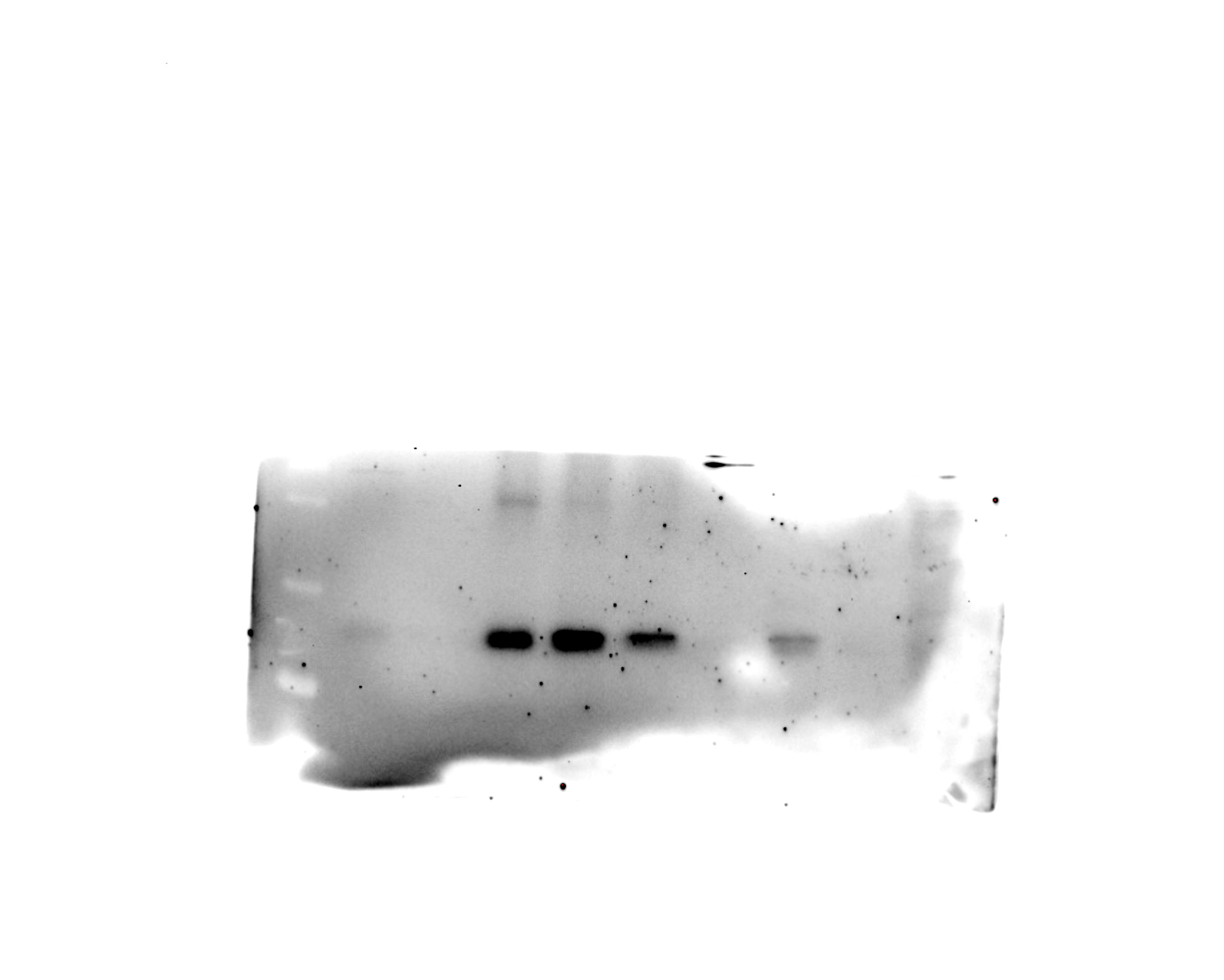


kDa

β-actin

p-RIPK3

Cl-Casp3

42

70

17

Cl-Casp1

20


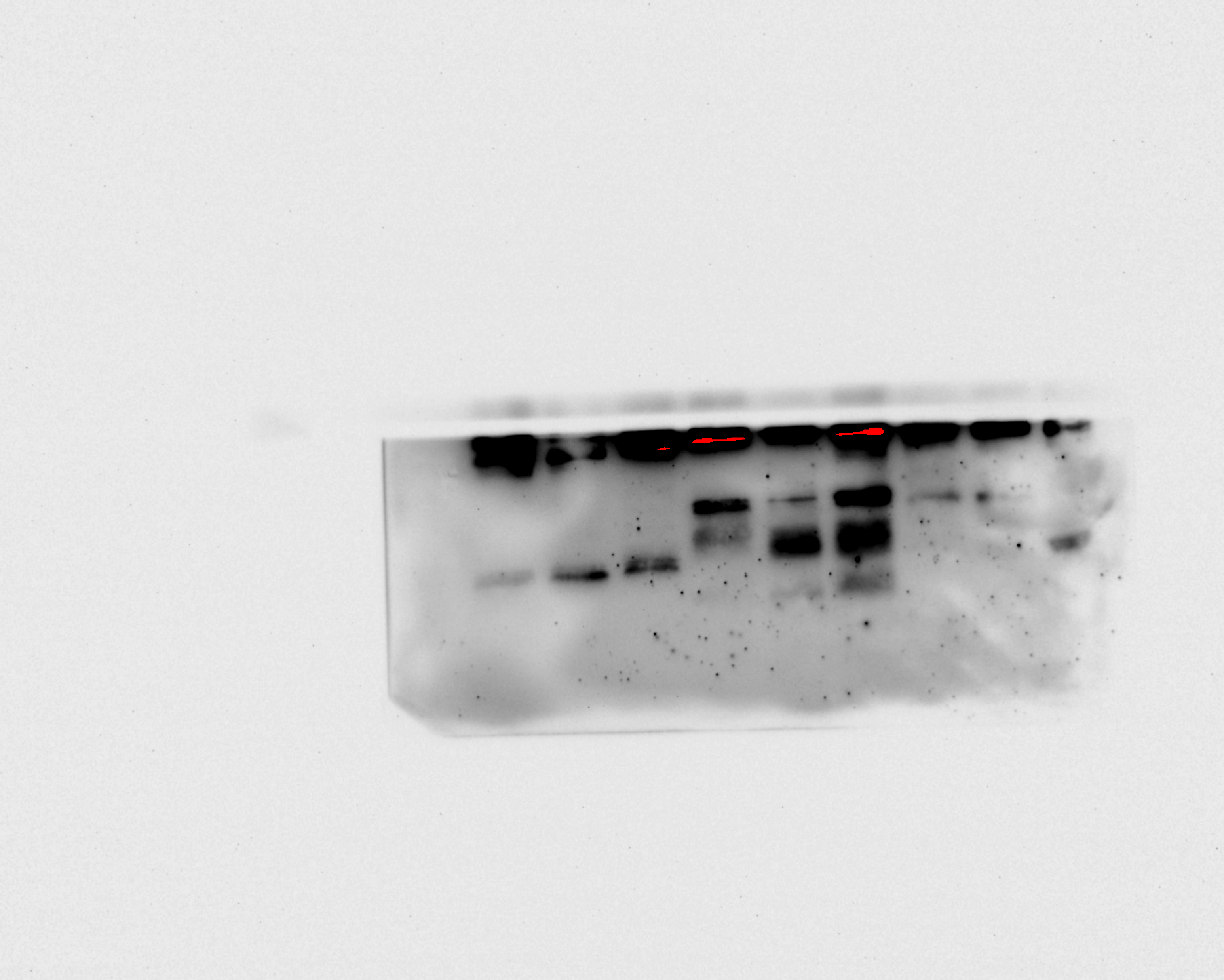

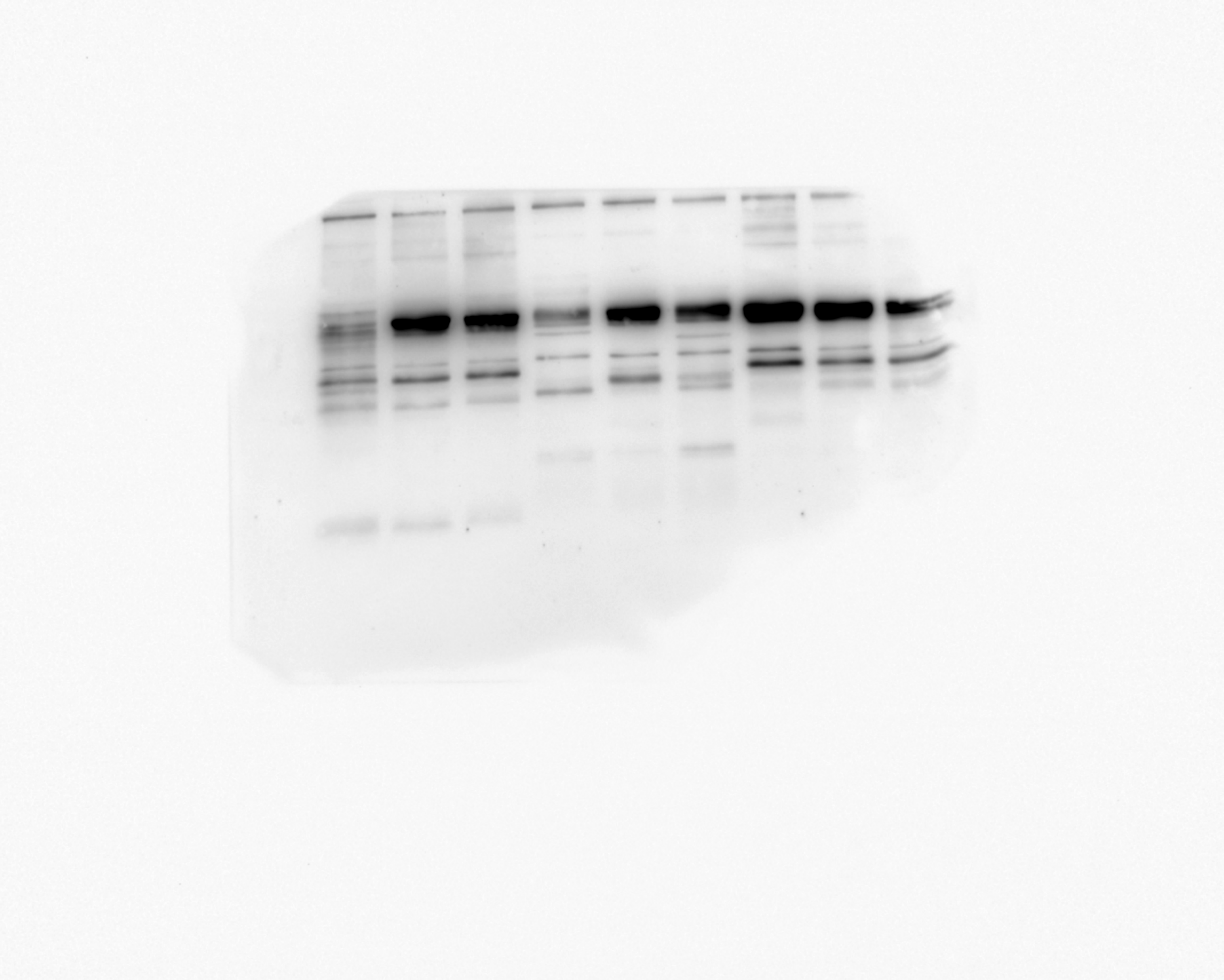


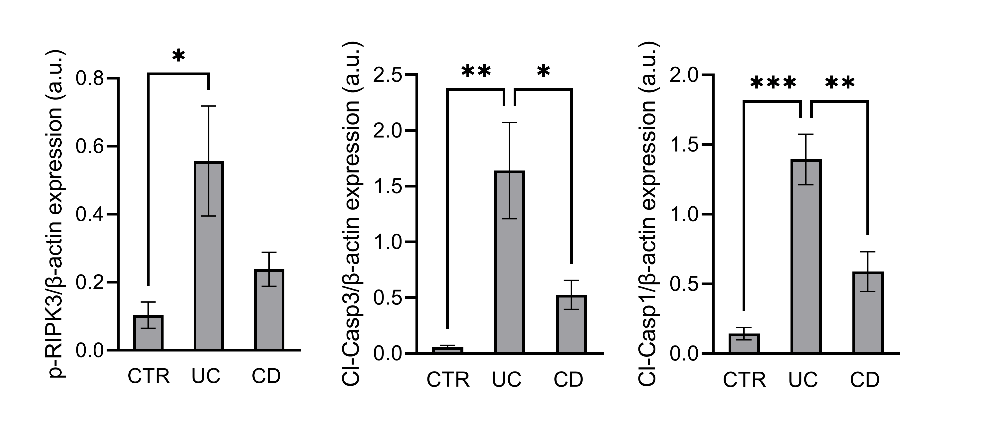


**Figure S1**. **PANoptotic markers are increased in the gut of ulcerative colitis patients.** Representative western blot showing active RIPK3 (phosphorylated RIPK3 or p-RIPK3), active caspase 3 (cleaved caspase 3 or Cl-Casp3), active caspase 1 (cleaved caspase 3 or Cl-Casp1) and β-actin expression in tissue lysates from colonic biopsies of two CTR, three patients with UC, and three patients with CD. Lower panels show the densitometric analysis of p-RIPK3/β-actin, Cl-Casp3/β-actin, and Cl-Casp1/β-actin ratio of the samples analyzed. Values are expressed as arbitrary units (a.u.). Results are shown as mean ± SEM. *p<0.05, **p<0.01, ***p<0.001; one-way ANOVA followed by Dunnett’s test.
